# Supplementary material for: Social distancing and preventive practices of government employees in response to COVID-19 in Ethiopia
Source: PLoS One. 2021 Sep 7;16(9):e0257112. doi: 10.1371/journal.pone.0257112 (PMC8423289; doi:10.1371/journal.pone.0257112)
Supplement: S1 Appendix — (PDF) [file pone.0257112.s001.pdf]

S1 Appendix. List of institutions/organizations included in the survey with collected samples, June 2020

No.	Government level<br>National Offices	Collected Sample (#)
1	Ministry of Health	50
2	Ministry of Education	50
3	Ministry of Science and Higher Education	30
4	Ministry of Finance	50
5	Ministry of Trade and Industry	35
6	Ministry of Labor and Social Affairs	30
7	Ministry of Women, Children and Youth	27
8	Ministry of Agriculture	46
9	Ministry of Transport	34
10	Ministry of Urban Development and Construction	41
11	Ministry of Innovation and Technology	29
12	Ministry of Culture and Tourism	40
12	Ministry of Water, Irrigation and Energy	37
14	Ministry of Revenues	40
15	National Bank of Ethiopia	41
16	Commercial Bank of Ethiopia (Addis Ababa Branch)	44
Subtotal	***16***	***624***
	**Addis Ababa City Administration Offices**	
1	Health Bureau	40
2	Education Bureau	39
3	Finance Bureau	40
4	Trade and Industry Bureau	30
5	Bureau of Labor and Social Affairs	30
6	Bureau of Women, Children and Youth	28
7	Bureau of Agriculture	26
8	Bureau of Transport	38
9	Bureau of Construction	29
10	Bureau of Culture and Tourism	30
11	Water and Sewerage Authority	25
12	Bureau of Revenues	28
13	Yeka Sub-City Administration Office	39
14	Lideta Sub-City Administration Office	40
15	Arada Sub-City Administration Office	30
16	Gulele Sub-City Administration Office	40
17	Bole Sub-City Administration Office	40
18	Addis Ketema Sub-city Administration Office	38
Subtotal	***18***	***610***
	**Oromia Regional State Offices**	
1	Health Bureau	58
2	Education Bureau	18
3	Finance Bureau	30
4	Bureau of Trade and Industry	21
5	Bureau of Labor and Social Affairs	33

6	B. of Women, Children and Youth	24
7	Bureau of Agriculture	33
8	Bureau of Transport	24
9	Bureau of Urban Development and Construction	26
10	Bureau of Culture and Tourism	21
11	Bureau of Water, Irrigation and Energy	21
12	Bureau of Revenues	30
Subtotal	***12***	***339***
**Overall total**	**46**	**1,573**
