# Supplementary material for: Social distancing and preventive practices of government employees in response to COVID-19 in Ethiopia
Source: PLoS One. 2021 Sep 7;16(9):e0257112. doi: 10.1371/journal.pone.0257112 (PMC8423289; doi:10.1371/journal.pone.0257112)
Supplement: S2 Appendix — (PDF) [file pone.0257112.s002.pdf]

S2 Appendix. English version self-administered questionnaire developed for the survey, June 2020

1. What is the name of your institution/ organization? ____

2. In which Sub-city of Addis Ababa is your institution located? ________________________________

3. In which Sub-city of Addis Ababa is your current residence? ________________________________
   If out of Addis Ababa, indicate the name of the woreda or city: ___________________________

4. What is your gender? 1. Male 2. Female

5. What is your age (years)? _______

6. What is the highest level of your educational qualification?
   1. Less than 12th Grade
   2. 12th Grade complete (only)
   3. Diploma (12th Grade complete and 1 or more years of training)
   4. Bachelor's Degree
   5. Master's Degree
   6. MD/Specialist
   7. PhD Degree
   8. Other (please describe) ______________________________________

7. How long (years of experience) have you served in this institution? ________

8. How many people live in your household, including yourself? _____________

9. Of the following COVID-19 preventive measures, which one have you currently applied to prevent your-self against COVID-19? (Circle all that apply)

9.1 Stay home	1. Yes	2. No
9.2 Maintain physical distancing	1. Yes	2. No
9.3 Avoid close contact with people including hand shaking	1. Yes	2. No
9.4 Cover mouth/nose with face/cloth mask when going outdoors	1. Yes	2. No
9.5 Frequent hand washing with water and soap	1. Yes	2. No
9.6 Avoiding touching your eyes, nose, and mouth with unwashed hands	1. Yes	2. No
9.7 Avoid mass gatherings	1. Yes	2. No
9.8 Covering your mouth and nose when you cough or sneeze	1. Yes	2. No
9.9 Limit movement	1. Yes	2. No
9.10 Disinfecting surfaces	1. Yes	2. No
9.11 Disinfecting the mobile phone	1. Yes	2. No
9.12 Eating garlic, ginger, lemon	1. Yes	2. No

   9.13 Another preventive measure, please specify ___________________________

10. Do you follow the recommendations from authorities in Ethiopia to prevent the spread of novel coronavirus?
   1. Do not follow at all
   2. Follow sometimes

3. Follow consistently always

11. Do you recommend the use of a facemask for people who are well and not in the healthcare settings?
    1. Strongly recommend
    2. Recommend
    3. Neither recommends nor not recommend
    4. Not recommend
    5. Strongly not recommend

12. Consistently wearing a facemask is highly effective in protecting you from getting infected with coronavirus.
    1. Strongly agree
    2. Agree
    3. Neither agree nor disagree
    4. Disagree
    5. Strongly disagree

13. Have you ever tested for novel coronavirus?

    1. Yes 2. No

14. How sure are you that you could get a novel coronavirus test if you want to be tested?
    1. Not at all sure
    2. Only a little sure
    3. Somewhat sure
    4. Very sure
    5. Completely sure
    6. Don't Know

15. Have you ever been quarantined due to COVID-19?

    1. Yes 2. No

16. Do you have any chronic illness? 1. Yes 2. No 3. Don't know 4. Not want to disclose

17. I think the policy decisions that have been made by the Government to contain the spread of the novel coronavirus are fair and reasonable.
    1. Strongly disagree
    2. Disagree
    3. Neither agrees nor disagrees
    4. Agree
    5. Strongly agree

18. In your opinion, what do you think about the adequacy of the current measures taken by the Government to contain the spread of COVID-19 epidemic in Ethiopia?
    1. Very inadequate
    2. Inadequate
    3. Neither adequate nor inadequate
    4. Adequate
    5. Very adequate

Thank you very much for your time!
