# Supplementary material for: Social distancing and preventive practices of government employees in response to COVID-19 in Ethiopia
Source: PLoS One. 2021 Sep 7;16(9):e0257112. doi: 10.1371/journal.pone.0257112 (PMC8423289; doi:10.1371/journal.pone.0257112)
Supplement: S3 Appendix — (PDF) [file pone.0257112.s003.pdf]

S3 Appendix. Amharic version of self-administered questionnaire used for the survey, June 2020

በአዲስ አበባ ከተማ የሚገኙ የመንግሥት ሠራተኞችን የኮሮና ቫይረስ በሽታ (ኮቪድ-19) የመከላከል ልምዶችን ለማጥናት የተዘጋጀ መጠይቅ (ግንቦት-ሰኔ 2012 ዓ.ም.)

1. የመሥሪያ ቤትዎ ስም፤ ______________________________

2. መሥሪያ ቤትዎ የሚገኝበት የአዲስ አበባ ክፍለ ከተማ ስም፤ ______________________________

3. በአሁኑ ጊዜ የመኖሪያ ቤትዎ የሚገኝበት የአዲስ አበባ ክፍለ ከተማ ስም፤ ______________________________
   ከአዲስ አበባ ዉጪ ከሆነ የወረዳዉን ወይም የከተማዉን ስም ይጻፉ፤______________________________

4. ጾታ 1. ወንድ 2. ሴት

5. እድሜ (በዓመት) --------------------

6. ያጠናቀቁት ከፍተኛው የትምህርት ደረጃ፤
   1. ከ12ኛ ክፍል በታች
   2. 12ኛ ክፍል ያጠናቀቀ (ብቻ)
   3. ዲፕሎማ (12ኛ ክፍል ያጠናቀቀ እና 1 ወይም ከዚያ በላይ የስልጠና ዓመት ያለዉ)
   4. የመጀመሪያ ዲግሪ
   5. ሁለተኛ ዲግሪ
   6. የሕክምና ዶክተር/ስፔሻሊስት
   7. ፒ. ኤች. ዲ ዲግሪ
   8. ሌላ ካለ እባክዎ ይጥቀሱ----------------------------------

7. በዚህ መሥሪያ ቤት ውስጥ ለምን ያህል ዓመት (የሥራ ልምድ) አገልግለዋል? -------------------------

8. እርስዎን ጨምሮ በአሁኑ ጊዜ አንድ ላይ የሚኖሩት የቤተሰብዎ አባላት ስንት ናቸው? ----------------------

9. ከሚከተሉት የኮሮና ቫይረስ በሽታ የመከላከያ ዜዴዎች ውስጥ እራስዎን ከበሽታው ለመከላከል በአሁኑ ጊዜ የትኛውን ዜዴ እየተጠቀሙ ነው? (ቀጥሎ ለተዘረዘሩት ሁሉ ይመልሱ)

23.1 በቤት ውስጥ መቆየት	1. አዎ	2. አይ
23.2 አካላዊ እርቀትን መጠበቅ	1. አዎ	2. አይ
23.3 የእጅ መጨባብጥን ጨምሮ ከሰዎች ጋር የቅርብ ግንኙነትን ማስወገድ	1. አዎ	2. አይ
23.4 ከቤት ውጪ በሚወጡበት ጊዜ አፍ/አፍንጫዎን የፊት ጭንብል /ጨርቅ መሸፈን	1. አዎ	2. አይ
23.5 በተደጋጋሚ እጅን በውሃ እና በሳሙና መታጠብ	1. አዎ	2. አይ
23.6 ባልታጠበ እጅ ዓይኖችዎን ፣አፍንጫዎን እና አፍዎን ከመንካት መቆጠብ	1. አዎ	2. አይ
23.7 የጅምላ ስብሰባዎችን ማሰወገድ	1. አዎ	2. አይ
23.8 በሚያስሉ ወይም በሚያስነጥሱ ጊዜ አፍዎን እና አፍንጫዎን መሸፈን	1. አዎ	2. አይ
23.9 እንቅስቃሴን መገደብ	1. አዎ	2. አይ
23.10 ሳኒታይዘር መጠቀም/ማፅዳት	1. አዎ	2. አይ
23.11 ተንቀሳቃሽ ስልክን በአልኮል ማፅደት	1. አዎ	2. አይ
23.12 ሎሚ፣ነጭ ሽንኩርት፤ዝንጅብል መመገብ	1. አዎ	2. አይ
23.13 ሌላ ካለ እባክዎ ይጥቀሱ -----------------------------------		

10. በእርስዎ አስተያየት የኮሮና ቫይረስ ሥርጭትን ለማቆም የኢትዮጵያ ባለሥልጣናት የሚሰጡትን ምክሮች ይከተላሉ?
    1. በጭራሽ አልከተልም
    2. አንዳንድ ጊዜ እከተላለሁ
    3. ሁል ጊዜ በቋሚነት እከተላለሁ

11. በጤና ተቋማት ውስጥ ላልሆኑ ጤነኛ ሰዎች የፊት ጭንብል (ማስክ) እንዲጠቀሙ ይመክራሉ?
    1. በጣም እመክራለሁ

2. እመክራለሁ
3. ገለልተኛ ነኝ
4. አልመክርም
5. በጭራሽ አልመክርም

12. የፊት ጭንብልን (ማስክ) በቋሚነት መልበስ በኮሮና ቫይረስ እንዳይያዙ ለመከላከል ውጤታማ ነው ብለዉ ያስባሉ?
    1. በጣም እስማማለሁ
    2. እስማማለሁ
    3. ገለልተኛ ነኝ
    4. አልስማማም
    5. በጣም አልስማማም

13. ለኮሮና ቫይረስ በሽታ ምርመራ አድርገው ያውቃሉ? 1. አዎ 2. አይ

14. ለኮሮና ቫይረስ ምርመራ ቢፈልጉ ምርመራውን ለማግኘት ምን ያህል እርግጠኛ ነዎት?
    1. በጭራሽ እርግጠኛ አይደለሁም
    2. ትንሽ እርግጠኛ ነኝ
    3. በተወሰነ ደረጃ እርግጠኛ ነኝ
    4. በጣም እርግጠኛ ነኝ
    5. ሙሉ በሙሉ እርግጠኛ ነኝ
    6. አላውቅም

15. በኮሮና ቫይረስ በሽታ ምክንያት በለይቶ ማቆያ ውስጥ ሆነው የውቃሉ? 1. አዎ 2. አይ

16. ማንኛውም ዓይነት ሥር የሰደደ በሽታ (chronic illness) አለብዎት?
    1. አዎ 2. አይ 3. አላውቅም 4. መግለጽ አልፈልግም

17. የኮሮና ቫይረስ ስርጭትን ለመግታት በመንግስት የተደረጉ የፖሊሲ ውሳኔዎች ትክክለኛ እና ምክንያታዊ ናቸው ብለዉ ያስባሉ?
    1. በጣም አልስማማም
    2. አልስማማም
    3. ገለልተኛ ነኝ
    4. እስማማለሁ
    5. በጣም እስማማለሁ

18. በርስዎ እምነት፤ የኮሮና ቫይረስ ወረርሽኝን ለመግታት መንግሥት በአሁኑ ጊዜ እየወሰደ ያለዉ እርምጃ በቂ ነዉ ብለዉ ያስባሉ?
    1. በፍጹም በቂ አይደለም
    2. በቂ አይደለም
    3. በቂም፤ አነስተኛም አይደለም
    4. በቂ ነዉ
    5. በጣም በቂ ነዉ

ስላደረጉት አስተዋጽኦ እና ውድ ጊዜዎን ስለሰጡን በጣም እናመሰግናለን!
