# Supplementary material for: Social distancing and preventive practices of government employees in response to COVID-19 in Ethiopia
Source: PLoS One. 2021 Sep 7;16(9):e0257112. doi: 10.1371/journal.pone.0257112 (PMC8423289; doi:10.1371/journal.pone.0257112)
Supplement: S4 Appendix — (PDF) [file pone.0257112.s004.pdf]

S4 Appendix. Afan Oromo version of self-administered questionnaire used for the survey, June 2020

Gaaffilee Shaakala Ittisa Dhibee Koronaa (COVID-19) Hojjettoota Mootummaa Finfinnee Keessa Hojjetaniif Qophaa'e (Caamsaa-Waxabajjii 2012 EC)

1. Maqaa mana hojii keessanii: ____
2. Maqaa kutaa Finfinnee (Sub-city) manni hojii keessatti argamu? ___________________________
3. Maqaa kutaa Finfinnee manni jireeynaa keessan keessatti argamu? _________________________
   - Teessoon mana jireeynaa kutaa Finfinnee ala yoo ta'e maqaa ibsaa: ___________________
4. Saala? 1. Dhiira 2. Dhalaa
5. Umurii (Waggaan)? __________
6. Sadarkaan barnoota keessan inni ol-aanaan kami dha?
   1. Kutaa 12^ffaa gad
   2. Kutaa kudha lama qofaa kan xumure
   3. Dipiloomaa (kutaa 12^ffaa kan xumuree fi wagga 1 fi isaa ol leenjii kan qabu/du)
   4. Digirii jalqabaa
   5. Digirii lammaffaa
   6. Hakiima/Ispeeshaalistii
   7. Digirii sadaffaa (PhD)
   8. Kan biroo (maaloo ibsaa) ______________________________________
7. Mana hojii kana keessa waggaa meeqa tajaajiltaniittuu (muxxannoo hojii)? ____________
8. Isinin dabalatee mana keessan keessa nama meeqa taatanii jiraattu? __________________
9. Ejjennoowwan/gochawwan dhibee vaayirasii koronaa ittisuu danda'an armaan gadiitti ibsaman keessa, dhibee kana irraa of eeguuf isin yeroo ammaa kana isa kamitti fayyadamtu? *(Barreffama hundaaf deebii kennaa)*

23.1 Mana turuu	1. Eeyyeen	2. Lakkii
23.2 Wal'irraa fageenya qaamaa eeggachuu	1. Eeyyeen	2. Lakkii
23.3 Tuttuuqqii namoota waliin qabnu harka walqabachuu dabalatee dhaabuu	1. Eeyyeen	2. Lakkii
23.4 Manaa yeroo bahanitti maaskii afaan/funyaan irratti godhachuu	1. Eeyyeen	2. Lakkii
23.5 Yeroo hunda harka bishaanii fi saamunaan sirritti dhiqachuu	1. Eeyyeen	2. Lakkii
23.6 Harka osoo hin dhiqatiin ija, funyaanii fi afaan tuttuquu dhiisuu	1. Eeyyeen	2. Lakkii
23.7. Bakka namoonni baay'atanii walitti qabaman dhaquu dhiisuu	1. Eeyyeen	2. Lakkii
23.8 Yeroo qufaatu ykn axxiffattu afaanii fi funyaan kee haguuggachuu	1. Eeyyeen	2. Lakkii
23.9 Sochii bakka adda addaa deemuu xiqqeessuu	1. Eeyyeen	2. Lakkii
23.10 Sanitaayizerii (dis-infectant) fayyadamuu	1. Eeyyeen	2.Lakkii
23.11 Moobaayilii sanitaayizeriin qulqulleessuu	1. Eeyyeen	2. Lakkii
23.12 Jinjibila (garlic), qullubbii adii fi loomii fayyadamuu	1. Eeyyeen	2. Lakkii

23.13. Ejjennoo/gochaawwan ittisa biro (maaloo ibsaa)_______________________________

10. Akka yaada keessaniitti, babal'achuu dhibee koronaa vaayiresii kana dhorkuuf dambiiwwanii fi qajeelfamoota akka biyyaatti bahan ni hordoftuu?
    1. Gonkumaa hin hordofu
    2. Yeroo tokko tokko nan hordofa
    3. Yeroo hunda haala itti fufinsa qabuunaan hordofa

11. Namoota fayya qabeeyyii ta'anii fi bakkeewwan tajaajilli fayyaa itti kennamu keessa hin jirreef maaskii fuullaa akka fayyadaman ni gorsituu?
    1. Baay'een gorsa
    2. Nan gorsa
    3. Gorsuu, gorsuu dhiisus nan danda'a
    4. Hin gorsu
    5. Baay'ee hin gorsu

12. Yeroo hunda maaskii fuulaa fayyadamuun dhibee koronaan akka hin qabamne sirritti ittisa yaada jedhu qabduu?
    1. Baay'een walii-gala
    2. Waliin-gala
    3. Waliin-galas walii-hingalus
    4. Walii-hingalu
    5. Baay'ee walii-hingalu

13. Dhibee vaayirasii koronaatiif qoratamtanii (laboratory) beektuu? 1. Eeyyeen 2. Lakkii

14. Osoo dhibee vaayirasii koronaatiif qoratamuu barbaaddanii qorannoo laaboratoorii nan argadha jettanii amantuu?
    1. Homaa hin amanu
    2. Xiqqo qofan amana
    3. Hanga tokko nan amana
    4. Baay'een amana
    5. Guutummaa guututtan amana
    6. Hin beeku

15. Sababa dhibee koronaa kanaan kan ka'e bakka turmaataa (quarantine) seentanii beektuu?
    1. Eeyyeen 2. Lakkii

16. Dhibee qancaroo (Chronic illness) ta'e qabduu?
    1. Eyyeen 2. Lakkii 3. Hin beeku 4. Deebisuu hin barbaadu

17. Ejjennoowwanii fi murteewwan babal'ina dhibee vaayiresii koronaa xiqqeessuuf mootummaadhaan darbanii hojiirra oolaa jiran sirriidhaa fi ta'uu kan qabudha jettanii yaadduu?
    1. Baay'ee walii hin galu
    2. Walii hin galu
    3. Waliingalas walii hin galus
    4. Waliingala
    5. Baay'een waliigala

18. Akka yaada keessanitti qabiyyeen ejjennoowwan mootummaadhaan ittisa weerara dhibee vaayiresii koronaa irratti fudhatamaa jiru ga'umsi isaa maal isinitti fakkaataa?
    1. Baay'ee gahaa miti
    2. Gahaa miti
    3. Gahaadhas gahaas miti
    4. Gahaa dha
    5. Baay'ee gaha dha

Yeroo keessan fudhattanii deebii waan waan nuu kennitaniif guddaa galatoomaa!
