# Supplementary figures and images for: Social distancing and preventive practices of government employees in response to COVID-19 in Ethiopia
Source: PLoS One. 2021 Sep 7;16(9):e0257112. doi: 10.1371/journal.pone.0257112 (PMC8423289; doi:10.1371/journal.pone.0257112)

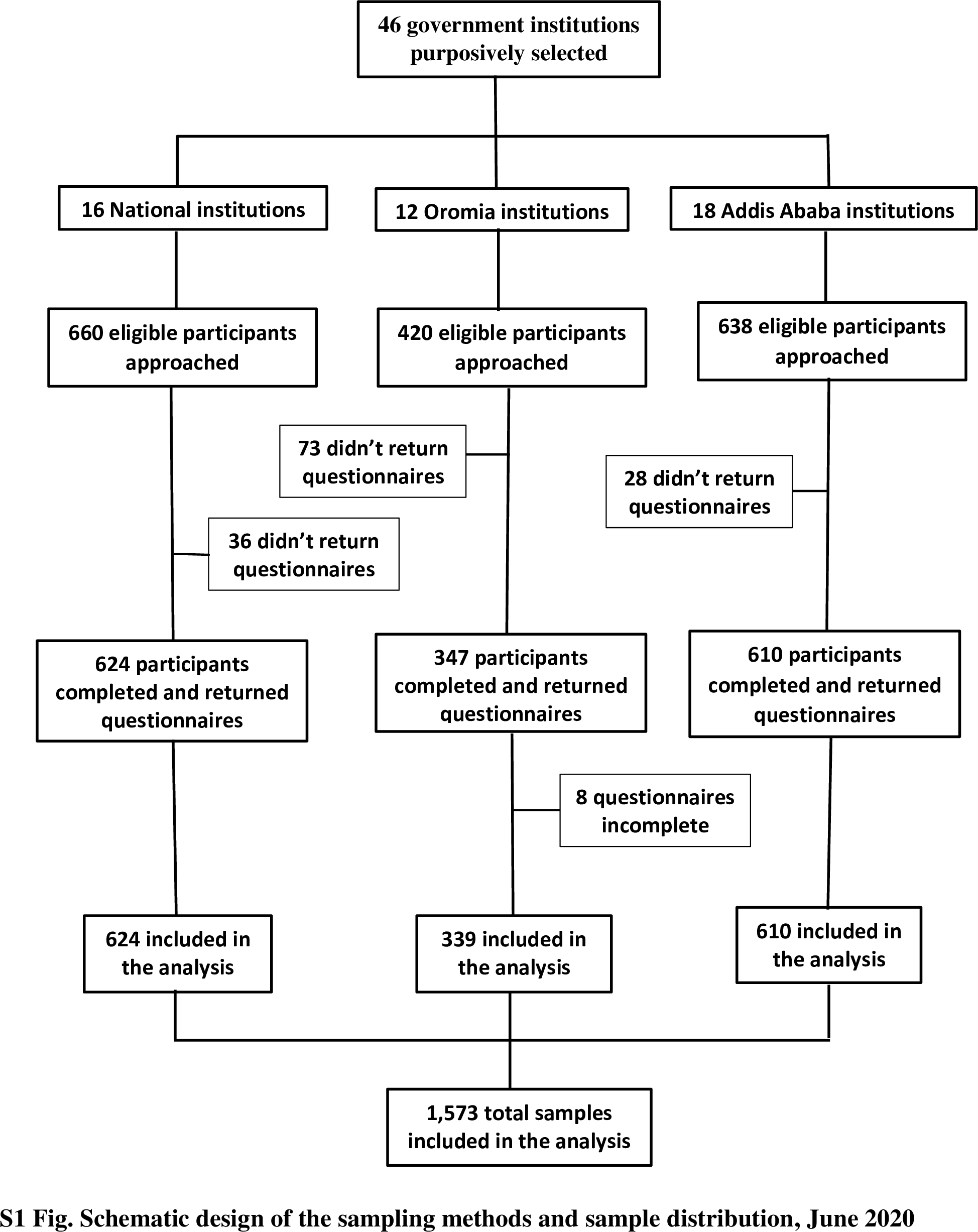

Supplement: S1 Fig — (TIF) [file pone.0257112.s005.tif]
