# Supplementary material for: Social distancing and preventive practices of government employees in response to COVID-19 in Ethiopia
Source: PLoS One. 2021 Sep 7;16(9):e0257112. doi: 10.1371/journal.pone.0257112 (PMC8423289; doi:10.1371/journal.pone.0257112)
Supplement: Attachment — Submitted filename: PONE-D-21-22047.docx [file pone.0257112.s007.docx]

**Comments to the authors:**

The present study deals with the impact of social distancing and other preventive behaviors of government employs in response to Covid-19. A cross-sectional study covering large number of individuals from different government institutions. The study implicates the importance of social measures in preventing the spread of COVID-19 in government employees. Although the study covers the large number of individuals there is no experimental proof to data and the study is mere observatory of know preventive factors. The authors also failed to explain the effectiveness of these measures in COVID-19 positive symptomatic and asymptomatic individuals. Overall, the manuscript cannot be considered for publication in present form and will require substantial improvement for consideration. The primary concern regarding the current manuscript is the Novelty. The following queries may be considered before submitting the revision.

1. Author should provide schematic diagram of study design for more clarity.
2. Novelty is missing in the study.
3. The authors should revise the discussion part thoroughly in the manuscript which
4. seems very weak to support the study.
5. References are missing at the relevant places. Previous studies describing the importance of face masking and social distancing in COVID-19 prevention should be cited
